# Caloric Restriction and Remission of Severe Chronic Spontaneous Urticaria: An Autobiographical Case Report

**DOI:** 10.7759/cureus.19371

**Published:** 2021-11-08

**Authors:** Mohammed Abrahim

**Affiliations:** 1 Emergency, Halton Healthcare, Milton, CAN; 2 Family Medicine, McMaster University, Hamilton, CAN

**Keywords:** urticaria, obesity-related illnesses, abdominal obesity, visceral fat percentage, chronic spontaneous urticaria, autobiographical case report, caloric restriction, weight loss and obesity, hives, chronic idiopathic urticaria

## Abstract

In this autobiographical case report, the physician-author-patient is documenting the remission of his own severe chronic spontaneous urticaria (CSU) in response to caloric restriction and subsequent weight loss. To my knowledge, this is the first reported case of CSU remission secondary to weight loss in the literature. CSU is a common debilitating pruritic skin condition that carries a significant economic and psychological burden. Currently, the mainstay of CSU treatment is symptom control, rather than seeking to achieve complete remission. Despite some recent retrospective studies reporting an association between obesity and CSU, there is a paucity of interventional research testing the impact of obesity management on CSU. The case reported herein highlights the need for research able to test the hypothesized association between obesity, particularly visceral obesity, and CSU.

## Introduction

Urticaria is a common skin condition characterized by the development of wheals (hives), angioedema, or both [1]. Furthermore, urticaria is classified based on its duration as acute (≤6 weeks) or chronic (>6 weeks). Urticaria is further classified as inducible (eliciting factor involved) or spontaneous (no eliciting factor) [1]. Chronic spontaneous urticaria (CSU) is the most common type of urticaria. The global prevalence of CSU is estimated to be between 0.5% and 1% [2].

The most common age of occurrence of CSU is between 20 and 40 years [2]. Because the age of diagnosis usually matches the peak work productivity years, the condition can have a significant economic impact because of missed workdays [2]. Furthermore, the extreme discomfort experienced by those living with CSU can have a significant impact upon the quality of life in addition to the adverse effects of high-dose, long-term antihistamine use [2]. Attempts have been made to create tools to objectively evaluate and standardize the burden of the disease. The recent international consensus guidelines recommended using urticaria activity score (UAS7) to assess CSU disease activity and treatment response both in clinical care and trials. The Urticaria Activity Score summed over seven days (UAS7) assesses the wheals count and pruritus severity in CSU using a patient-reported daily diary. UAS7 values range from 0 to 42, with higher values reflecting higher disease activity and severity [1].

To date, there is no clear understanding of CSU pathogenesis, resulting in a lack of therapeutic efficacy [1]. The current treatment guidelines are directed primarily toward symptom management rather than complete remission of the disease. Second-generation H1-antihistamines are considered the first line of treatment [1]. In patients with CSU unresponsive to this initial therapy, dose increase is recommended as the second-line therapy up to a maximum of fourfold before other treatments are considered. For cases refractory to high-dose antihistamines, referral to an allergist or immunologist is recommended in order to initiate further pharmacological therapy, such as omalizumab [1]. Patients with CSU who do not show sufficient benefit from treatment with omalizumab at the recommended dose can be treated with omalizumab at higher doses, shorter intervals, or both [1]. Ciclosporin remains an optional therapy for the treatment of patients with CSU unresponsive to high dose second-generation H1-antihistamine and omalizumab [1]. Additionally, acute exacerbation of CSU requires a short course of systemic glucocorticoids [1]. Even with maximizing pharmacological therapy, more than half of patients living with CSU remain symptomatic [2].

Understandably, the lack of efficient CSU therapy leads to a great deal of frustration for patients and the treating physician alike [2]. Mast cell activation and the resultant histamine release play a major role in the expression of disease symptoms [3]. However, the cause of mast cell activation remains unclear. An autoimmune process has been proposed as potential pathogenesis [3]. Although CSU is not an allergic reaction, it can be confusing to patients, as well as treating physicians, because Urticaria, especially with angioedema, can be signs of life-threatening allergic reactions [1]. The dietary impact on CSU has been tested for involvement in the pathogenesis of CSU; however, several elimination diets are controversial and, as yet, unproven in well-designed double-blind placebo-controlled studies [3].

The physician author herein shares his clinical experiences as a CSU patient and his observations - be they coincidental or bona fide - regarding CSU. This case report is also evidently subject to all other limitations of single-case reports.

## Case presentation

**Figure 1:**
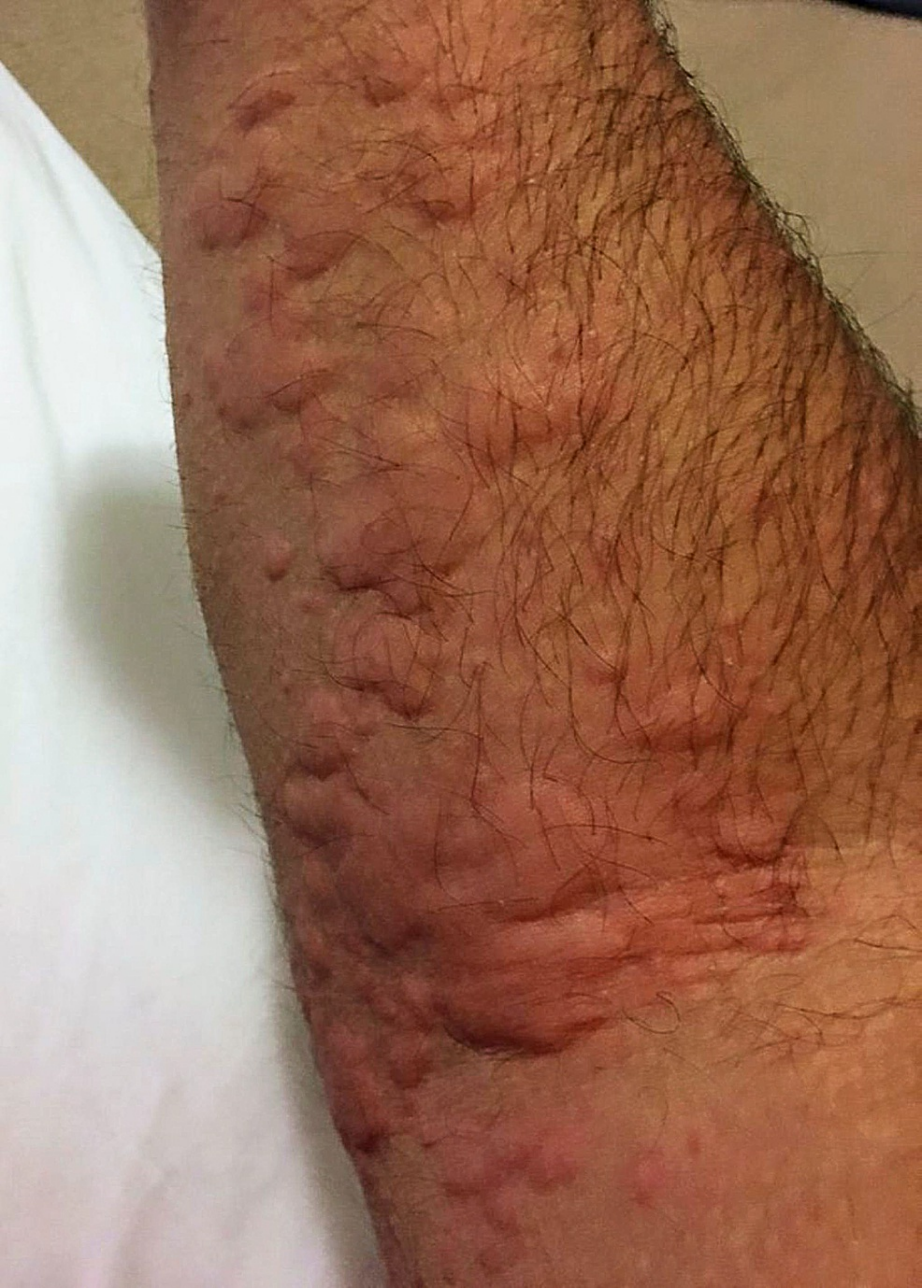
Urticarial wheals (hives) on the arm.

A 40-year-old man had episodes of urticaria beginning at age 12 years. The urticarial wheals were numerous and occurred on a daily basis with large confluent areas (Figure 1).

Moreover, angioedema of lips and eyelids occurred occasionally. He found the pruritus to be severe enough to interfere with normal daily activities, especially sleep. Interestingly, the onset of wheals was observed to be concurrent with the onset of prepubertal obesity. The initial diagnosis by his primary care physician was believed to be an allergic reaction. Because of this, he was initiated on second-generation H1-antihistamines, terfenadine 60 mg PO BID on a PRN basis for symptom control concurrently with attempting multiple elimination diets. However, the symptoms persisted for months. Eventually, the patient underwent several skin-prick tests to explore potential allergens, however, the results were repeatedly negative. Of note, the patient was also found to be IgA deficient. Although terfenadine was introduced as the first non-sedating antihistamine, the patient experienced sedation, confusion, and fatigue that was severe enough to interfere with his academic performance at medical school. It was often the case that the patient had to choose between the intense pruritus of the wheals or the lethargic complications of the treatment. Sometimes, the ailment was more tolerable than the treatment. Although the patient required daily antihistamines, the condition persisted for months. Subsequently, the patient was referred to the care of a dermatologist and an immunologist, with the condition eventually diagnosed as CSU. 

The CSU persisted throughout adulthood and occurred daily. The number of wheals was about 30 wheals per day which were intensely pruritic that interfered with daily functions especially sleep. The CSU was diagnosed as severe based on his UAS7 score of 35. The CSU demonstrated poor response to high-dose second-generation H1-antihistamines (cetirizine 20 mg PO TID) and occasional severe flare-ups that were refractory to high-dose antihistamines. In these instances, the patient required initiation of short-term glucocorticoid prescriptions (prednisone 50 mg PO daily for five days). Rarely (about once a year), the patient (now an emergency physician) visited the Emergency Department (ED) for severe urticaria and angioedema flare-ups which required occasional Epinephrine injections, some of those visits took place while working in the ED and being cared for by co-workers; this crossover relationship between physician and patient was, at times, embarrassing. He also used to wear long-sleeve shirts under the scrubs to cover up as many of the hives as possible. On the other hand, angioedema of the lips was easily covered by wearing a mask.

**Figure 2:**
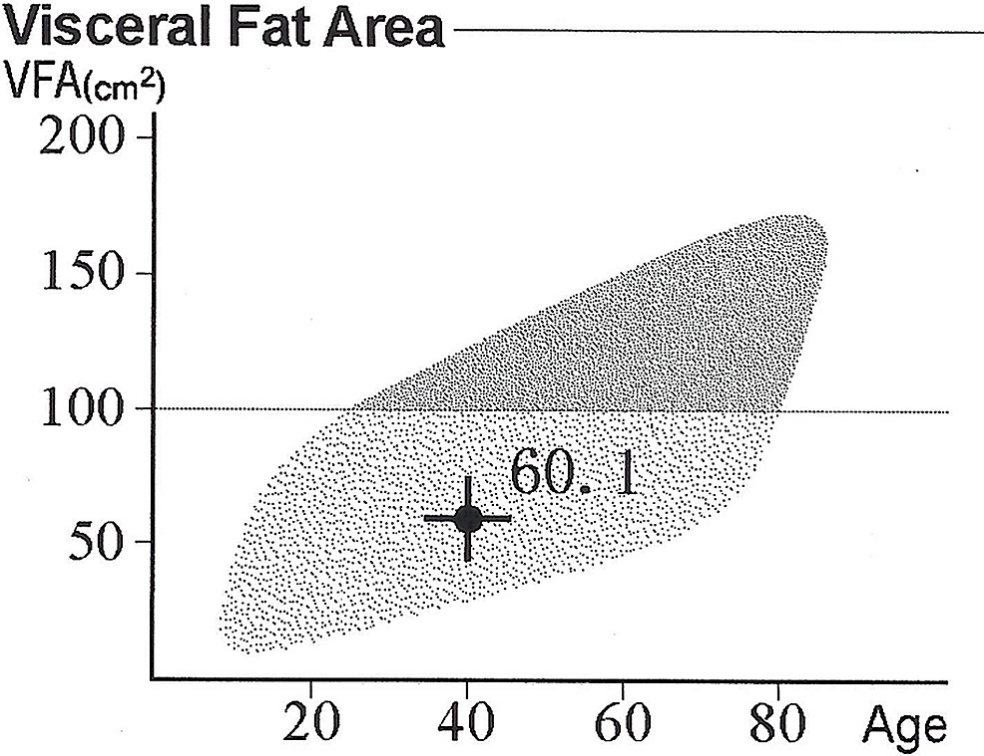
Visceral fat volumetric assessment after weight loss

The patient was eligible for biologic therapy; however, due to his previous experience with medication adverse events and lack of disease control, he declined that option. At 40, the patient weighed 120 kg (265 lb) at a height of 180 cm (5ft, 11in) - resulting in a BMI of 37 (class II obesity category). Furthermore, his waist circumference was 122 cm (47 in). He also suffered from obesity-related metabolic syndrome. Since the onset of obesity in childhood, the patient attempted multiple dieting plans which achieved only short-term weight loss followed by weight regain. In an attempt to manage the metabolic complications of obesity, he adopted a long-term plan of weight loss through caloric restriction without elimination of any food category. The plan consisted of losing about 0.5-1 kg (1-2 lb) per week via consumption of foods with low caloric density, such as whole plants and lean meat/fish, and reducing (not eliminating) calorie-dense foods such as oils, butter, visible animal fat, sugars, and flours. The total daily caloric intake ranged between 1,400 and 1,600 kcal/day. He initially lost about 30 kg over the duration of six months. Following that, weight loss became slower approaching a normal BMI. In 2016, after eight months, his body weight reached about 80 kg (180 lb); equating to a BMI of 24.7 kg/m^2^ and a waist circumference of 85 cm. He obtained a body composition analysis (bioelectrical impedance analysis InBody 770) (Table 1), which demonstrated a low visceral fat volume for age (Figure 2).

**Table 1:** Body composition after weight loss

	Values	Total Body Water	Lean Body Mass	Weight
Intracellular Water (lbs)	68.8	112.0	153.4	180.2
Extracellular Water (lbs)	43.2			
Dry Lean Mass (lbs)	41.4			
Body Fat Mass (lbs)	26.7			

 

Although the primary goal of weight loss was the management of metabolic syndrome, the patient also observed the gradual resolution of CSU in terms of shorter duration accompanied by a requirement for smaller doses of antihistamines. He noticed that smaller doses of antihistamines controlled the symptoms, therefore, he tapered the dosage of cetirizine from 20 to 10 mg, and finally five daily for about three months. Following that, the wheals were reduced in number and frequency to occasional rather than a daily occurrence with a reduction in the intensity of the pruritus. After six months, and with losing about 40 kg, disease severity improved from severe (UAS7 = 35) to mild (UAS7 = 10). Following that, the patient only required 5 mg of cetirizine once or twice per week on a PRN basis without the need for corticosteroids due to the absence of angioedema or severe flareups. After eight months, the patient lost a total of 45 kg and he noted that the UAS7 fell gradually from 10 to 4 and then to 0, and he has remained in remission (UAS 7 score 0) for eight months. Subsequently, antihistamines were discontinued. The patient’s body weight fluctuated in the following years; of note, urticarial hives waxed and waned concordantly even with minor bodyweight fluctuations (3-6 kg weight regain). Although caloric restriction is not currently included in the treatment guidelines for CSU, the patient maintained caloric restriction and considered that as the primary treatment of CSU with ongoing positive results.

## Discussion

A limited amount of emerging research has revealed an association between obesity and CSU [4-6]. However, to date, no well-designed interventional studies have tested the impact of obesity treatment on CSU duration or severity. The first observation of the potential association between obesity and CSU was noted by Lapi et al. in 1996 in a nationwide Italian population-based study [4]. The epidemiological survey of adults observed that the risk of CSU was significantly increased among those living with obesity.

In 2020, Zbiciak-Nylec et al. enrolled 85 consecutive adult patients with CSU conducted presenting to a Polish dermatology center [5]. This study revealed a statistically significant association between CSU and obesity. Moreover, the study also observed a dose-response effect - the higher the BMI value, the longer the disease duration and greater surface area of the body affected.

Pediatric CSU has also demonstrated an association with obesity. In 2021, Staubach et al. conducted a retrospective case-control study of epidemiological routine data derived from a German health insurance company [6]. The study involved 2.3 million insured individuals, under 18 years of age, diagnosed with urticaria. Insured persons (<18 years) without a diagnosis of urticaria were selected as controls. The study revealed that obesity occurred more frequently in children with urticaria when compared with controls.

Some commentators argue that obesity is poorly defined. Although obesity refers to excess body fat, visceral fat, rather than the total amount of body fat, is linked to a state of chronic systemic inflammation [7]. Furthermore, visceral adipose tissue is biologically active with marked elevation of pro-inflammatory adipokines such as interleukins-6 (IL-6), tumor necrosis factor-alpha (TNF-α), and c-reactive protein (CRP) [7]. Interestingly, CSU also demonstrates a similar elevation of proinflammatory biomarkers with a suggested autoimmune mechanism in its pathogenesis [3]. A possible mechanism to explain the association between obesity and CSU is the concurrent elevation of systemic inflammatory markers in visceral obesity and CSU. Additionally, emerging evidence demonstrates that waist circumference, an indirect measure of visceral fat, may have better predictive utility in understanding factors associated with CSU duration compared with total body fat [8].

The goal of obesity treatment is fat loss. Furthermore, losing body fat via caloric restriction creates a negative energy balance, leading to fat loss through lipolysis [9]. The rate of lipolysis is higher in visceral, rather than subcutaneous, fat and lowest in the femoral/gluteal region [10]. Therefore, it is important to note that caloric restriction preferentially depletes the visceral fat compartment first due to its high rate of lipolysis [10].

In addition to the other metabolic benefits observed during calorie reduction, visceral fat reduction leads to a significant improvement in inflammatory and immune markers [9]. However, when fat loss is derived via the removal of subcutaneous fat via liposuction, no improvement in inflammatory markers is noted [11] - when surgeons performed large volume liposuction (20%-40% of excess body fat), mostly from the abdomen [11]. This observation demonstrates the stark difference in impact between visceral and subcutaneous fat loss.

Waist circumference is a convenient, yet indirect, measure of visceral fat [12]. However, the gold standard modality for direct volumetric measurement of visceral fat is Computerized Tomography (CT). However, its clinical utility is limited due to the high dose of ionizing radiation [12]. MRI use in clinical practice is considered safe. However, MRI is costly and not accessible to many health regions worldwide. Bioelectrical impedance analysis (BIA) is an emerging technique of body composition analysis. The patient detailed within the current case report used it due to its consumer availability. However, while gaining utility within research, this modality is not readily available to clinicians [12]. Finally, it is crucial to note that, in the context of CSU, it is essential to differentiate intentional from non-intentional weight loss. Non-intentional weight loss in CSU could be a sign of underlying conditions, including malignancies [4].

One must point out some limitations in the current case report. First, there is an absence of pre-interventional volumetric visceral fat assessment. However, using waist circumference is an accepted, indirect, measure of visceral fat in medical practice. Second, although the patient used corticosteroids for short durations a few times per year, the impact of corticosteroids on visceral adiposity cannot be ignored and should be considered as a potential confounding variable in the assessment of visceral fat volume in patients with CSU.

To date, there are no interventional studies in the literature that have tested the impact of weight loss, via caloric restriction, on CSU. Similar interventions of weight loss via caloric restriction, combined with exercise, lead to the reduction of visceral fat mass, improvements in asthma control, and reduced inflammatory markers and systemic inflammation [13]. The author herein observes that caloric restriction, and its consequent weight loss, could have a favorable impact on CSU - with the potential to put the disease in remission. To test this hypothesis, undertaking interventional and prospective research that, able to measure and assess the impact of weight loss via caloric restriction upon CSU, as well as anthropometric measurements of body composition before and after the suggested intervention may also elucidate a causal association and further our understanding of disease etiology.

## Conclusions

The author (who is also a physician and the patient) shares a novel observation of the complete remission of his own severe CSU following weight loss via caloric restriction. Although a single case report cannot provide a clear demonstration of an association or causality, the reported case highlights the urgent need for interventional research that can test the impact of weight loss, via caloric restriction, on the course and severity of such a disabling and poorly understood chronic disease.
